# Altered Functional Connectivity Differences in Salience Network as a Neuromarker of Suicide Risk in Euthymic Bipolar Disorder Patients

**DOI:** 10.3389/fnhum.2020.585766

**Published:** 2020-11-13

**Authors:** Anna Maria Sobczak, Bartosz Bohaterewicz, Tadeusz Marek, Magdalena Fafrowicz, Dominika Dudek, Marcin Siwek, Anna Tereszko, Anna Krupa, Amira Bryll, Adrian Andrzej Chrobak

**Affiliations:** ^1^Department of Cognitive Neuroscience and Neuroergonomics, Institute of Applied Psychology, Jagiellonian University, Kraków, Poland; ^2^Department of Psychology of Individual Differences, Psychological Diagnosis, and Psychometrics, Institute of Psychology, University of Social Sciences and Humanities, Warsaw, Poland; ^3^Department of Adult Psychiatry, Jagiellonian University Medical College, Kraków, Poland; ^4^Department of Affective Disorders, Jagiellonian University Medical College, Kraków, Poland; ^5^Chair of Psychiatry, Jagiellonian University Medical College, Kraków, Poland; ^6^Chair of Radiology, Jagiellonian University Medical College, Kraków, Poland

**Keywords:** neuroimaging, functional connectivity, resting state, bipolar disorder, suicide, euthymia, suicidal risk, fMRI

## Abstract

**Objective:**

The occurrence of death by suicide in patients diagnosed with bipolar disorder is as much as 60 times greater than in the general population. Even during the state of euthymia patients are characterized by suicide risk. The aim of the study is to investigate the baseline brain activity in euthymic bipolar disorder patients in regard to suicide risk. We hypothesized that patients compared to healthy control group will demonstrate altered functional connectivity among resting state networks which will be directly related to current suicide risk.

**Method:**

41 subjects were enrolled in the study consisting control group (*n* = 21) and euthymic bipolar disorder patients group (*n* = 20). Functional magnetic resonance imaging was used to evaluate resting state brain activity and ROI–ROI functional connectivity analysis was performed. Suicidal risk was estimated using The Suicide Behaviors Questionnaire-Revised.

**Results:**

A two sample *t*-test revealed decreased functional connectivity between regions involved in the salience network in patients compared to the control group. This decrease was negatively correlated with current suicide risk.

**Conclusion:**

Obtained results suggest the association between risk of suicide and activity of regions responsible for functions such as learning from mistakes, prospective thinking, and sensory integration.

## Introduction

It is estimated that one million suicides are committed each year (Sears et al., 2013; World Health Organization (WHO), 2016). They constitute more than a half of violent deaths worldwide – that said, it is a far more common cause of death than from crimes and war combined (Van Heeringen, 2018), which makes suicide a major public health problem.

Bipolar disorder (BD) is a recurrent chronic disorder affecting 1–3% of the world’s population, characterized by fluctuating mood episodes related with functional impairment (Weinstock and Miller, 2008) such as depression, mania, or mixed episodes (Judd et al., 2002). The remaining periods in which affective symptoms are not present are described as euthymic. According to studies, most people who commit suicide suffer from affective disorders (Gonda et al., 2012; Pompili et al., 2013) and as stated by da Silva Costa et al. (2015), the occurrence of death by suicide in patients diagnosed with BD is as much as 60 times greater in comparison to the general population, and even 30–50% of these patients make decision to attempt suicide at least once in their lifetime (Schaffer et al., 2015). In addition, when patient is not under treatment during euthymic state, a suicide risk is even higher (Gibbons et al., 2009). Previous studies revealed a link between general sleep disturbances and suicidal ideation, as well as other suicide related behaviors in BD patients during the state of euthymia (Rocha et al., 2016). Furthermore, Malloy-Diniz et al. (2011) showed that BD patients during euthymic state are more impulsive than healthy controls (HCs) while Parmentier et al. (2012) found out that these patients may experience indirect hostility and irritability associated with suicidal behaviors. What is more, BD patients during the euthymic state are less likely to be supervised by clinicians which increase the likelihood of oversight possible suicidal intent in this group of patients. In a recent years there is a growing interest in the use of functional magnetic resonance imaging (fMRI), as it is considered a promising and non-invasive technique enabling to identify particular brain circuits potentially responsible for suicidal behavior. Resting-state fMRI (rsfMRI) has been widely used in studying networks alterations in clinical settings, among others in those related with suicidal thoughts and behaviors among patients with BD and other affective disorders (Kang et al., 2017; Ambrosi et al., 2018; Fu et al., 2019). One of the most consistent results is the one showing decreased functional connectivity in default mode network (DMN) in BD patients as compared to HCs (Mamah et al., 2013; Meda et al., 2014; Wang et al., 2016). DMN, also known as the task-negative network (Fox et al., 2005) includes brain regions, with coherent activity during daydreaming or mind-wandering, when the subject is not focused on a particular task (Shulman et al., 1997; Raichle et al., 2001; Simpson et al., 2001; Mckiernan et al., 2003; Buckner et al., 2008). Aforementioned network involves the medial prefrontal cortex (mPFC), the inferior parietal lobe (iPL) and the posterior cingulate cortex (PCC; Buckner et al., 2008). Above regions participate in self-referential processing (Gusnard and Raichle, 2001), such as thinking about ourselves, remembering, and recalling the past, imagining and making plans for the future (Buckner et al., 2008; Spreng, 2012; Meyer and Lieberman, 2018), as well as social interactions (Gweon and Saxe, 2013). Noteworthy, Minzenberg et al. (2015) revealed that suicide behaviors among BD patients may be associated with increased activation in DMN. Additionally, authors demonstrated increased functional connectivity in the salience network (SN) and increased activation in BD patients in comparison to patients without suicide risk (Minzenberg et al., 2015). Moreover, further studies point on the fact that the altered activity of the regions involved in DMN and SN networks are associated with the suicide risk in mood disorders (Malhi et al., 2019b; Schwartz et al., 2019). The SN is a brain network involved in detecting and filtering salient stimuli (Menon and Uddin, 2010). It includes the anterior insula (AI), rostral prefrontal cortex (rPFC), anterior cingulate cortex (ACC), and supramarginal gyrus (SMG). SN is associated with cognitive and attention control, modulation of behavior, plays key role for sensory input (Peters et al., 2016) and have rich connections with other resting state networks. Self-awareness, social skills and communication are strong related with SN functioning as well (Menon, 2015). Alterations to SN activity are considered to have clinical consequences (Uddin, 2016) and in the recent study Gong et al. (2019) revealed decreased functional connectivity of the SN in BD patients compared to HCs. New studies (Van Heeringen, 2018; Malhi et al., 2019a) promote getting more knowledge about neurobiology of suicide related behaviors in order to identify specific biomarkers that could inform efforts to prevent suicide. Our objective is to examine baseline brain activity associated with suicide behaviors among euthymic BD patients in comparison with HCs. We formulated two hypothesis: (1) euthymic BD patients when compared to HCs will demonstrate altered functional connectivity in DMN, and the FC values will be significantly correlated with the severity of suicidal risk, and (2) the euthymic BD patients hen compared to HCs will display altered functional connectivity in the SN and the FC values will be significantly correlated with the severity of suicidal risk.

## Materials and Methods

### Participants

Forty-one subjects were enrolled in the study. Euthymic BD patients (*n* = 20) were recruited by psychiatrists and diagnosed according to DSM-5 and ICD-10 criteria. Scoring <11 points on the Montgomery–Asberg Depression Rating Scale (MADRS; McDowell, 2006) and <5 points on the Young Rating Scale for Mania (YMRS; Young et al., 1979) allowed classifying participant as euthymic. The Suicide Behaviors Questionnaire-Revised (SBQ-R) was used to measure current suicide risk in both groups. It is a self-report scale enabling suicidal tendencies assessment (Osman et al., 2001) while discriminating between suicidal and non-suicidal subjects based on cut-off score (Batterham et al., 2015). Inclusion criterion was treatment with valproic acid and antipsychotic drugs from the dibenzoazepine group: quetiapine, olanzapine, clozapine. In BD group participants had similar duration of treatment in order to provide a comparable profile of side effects. Exclusion criteria included: contraindications for fMRI; lithium treatment and treatments other than those aforementioned; severe, acute or chronic somatic, and neurological diseases; severe personality disorders; history of drug or alcohol abuse; injuries; diseases; history of eye surgery. Additional criteria for the control group were a diagnosis of mental illness or the history of mental illness in first-degree relatives. All participants were right-handed. The HC group (*n* = 21) were age- and gender-matched with BD patients (Table 1). Due to head movements interfering with the analysis of fMRI data, two subjects from the BD group and three subjects from the HC group were excluded. Finally, 18 BD patients and 18 HC were analyzed. The study was approved by the Jagiellonian University Bioethics Committee.

**TABLE 1:** The description of study groups.

	**BD group**	**HC group**	***P*-values**
Age [years, mean (SD)]^a^	36 (6.4)	35 (10.2)	0.420
Sex (men/women)^b^	7/11	9/9	0.411
BD type (I/II)	9/9	–	
Number of BD patients with history of psychotic symptoms	5	–	
Duration of treatment [years, mean (SD)]	6.6 (6.1)		
Number of affective episodes [mean, (SD)]	9.6 (11.0)		
Number of hypomanic episodes [mean, (SD)]	1.1 (1.4)		
Number of manic episodes [mean, (SD)]	2.7 (5.7)		
Number of depressive episodes [mean, (SD)]	5.8 (6.5)		
Mean head motion^a^	0.077 (0.036)	0.073 (0.039)	0.756
Medication			
	Number of patients (%)	Dose [mean mg (SD)]	
Quetiapine	6 (32%)	367.7 (233.8)	
Olanzapine	7 (37%)	9.6 (4.7)	
Valproic acid	10 (53%)	980 (315.5)	

*^*a*^*t*-test. ^*b*^Chi-square test. BD, bipolar disorder; HC, healthy controls; SD, standard deviation. Head motions in millimeters were evaluated according to the frame-wise displacement criteria by Van Dijk et al. (2012).*

### MRI Data Acquisition

MRI data were acquired with the use of a 3T Siemens Skyra MR System (Siemens Medical Solutions, Erlangen, Germany). Anatomical images were obtained using a sagittal 3D T1-weighted MPRAGE sequence with TE = 3.9 ms and TR = 2300 ms. Thirteen-minute functional resting-state (rsfMRI) BOLD images were acquired using a gradient-echo single-shot echo planar imaging sequence with the following parameters: FOV = 256 mm; TE = 27 ms; TR = 2060 ms; slice thickness = 3 mm; voxel size = 3 × 3 × 3 mm, with no gap. A total of 39 interleaved transverse slices and 400 volumes were acquired. During resting state procedure, subjects were instructed to think of nothing in particular, keep their eyes open, and not to fall asleep, which was controlled using an infrared binocular eye tracker (Ober Consulting). Due to the limited access to the MRI scanner, subjects were tested at the different times of the day ranging between 12 pm to 9 pm.

### Imaging Data Preprocessing

Raw data (Nifti format) preprocessing was performed using SPM12 software (Friston et al., 1994) and MATLAB v. 2018a (The MathWorks, Inc., Natick, MA, United States). The first 10 time points of the data were discarded due to signal equilibration. Preprocessing was carried out at 390 time points for every subject. Processing steps involved slice timing [number of slices = 39; reference slice = 19; slice order = (1:2:39, 2:2:38); interpolation 4 B-Spline] and realigning. Twelve rigid-body parameters were estimated and the ART-based software package was used in order to identify all the outlier scans. Head movements in one or more of the orthogonal directions above 3 mm or with a rotation above 3° (BD = 2, HC = 3) discarded subjects from further analysis. The averaged functional EPI image was coregistrated and overlapped with the T1 image. Functional images were converted to Montreal Neurological Institute (MNI) space using the standard EPI template in SPM 12 (SPM12; Wellcome Trust Centre for Neuroimaging, UCL, London, United Kingdom) and spatially resampled to 3 × 3 × 3 mm voxel size. For the 12 motion parameters derived from the realignment step, white matter and cerebrospinal fluid signals were removed by a linear regression. The global signal was included according to its potential to provide valuable information (Liu et al., 2017). In order to reduce low-frequency drift and high-frequency noise, the signal was band-pass filtered (0.01–0.08 Hz).

### Functional Connectivity Analysis

First level functional connectivity analysis was performed using an ROI-to-ROI approach. Regions of interests (ROIs) were chosen based on the Harvard-Oxford Atlas and localized in regard to their coordinates on *x*, *y*, and *z*-axes. Raw time courses were extracted from each subject using “ROI Signal Extractor” module in Data Processing & Analysis for Brain Imaging (DPABI) V4.3 (Yan et al., 2016) working under MATLAB version R2018a and SPM 12. Correlation matrix consisting of bivariate Pearson correlation coefficients were created (“corrcoef” function). Afterward, raw correlation values were transformed into Fisher *Z*-scores for normalization purposes (“zscore” function). The SN was defined with the following ROIs: anterior cingulate cortex (ACC; 0, 22, 35), left anterior insula (AInsula; −44, 13, 1), right anterior insula (AInsula; 47, 14, 0), left rostral prefrontal cortex (rPFC; −32, 45, 27), right rostral prefrontal cortex (rPFC; 32, 46, 27), left supramarginal gyrus (SMG; −60, −39, −31), and right supramarginal gyrus (SMG; 62, −35, 32). The DMN was defined with the following ROIs: medial prefrontal cortex (mPFC; 1, 55, −3), left parietal lobe (LP; −39, −77, 33), right parietal lobe (LP; 47, −67, 29), and posterior cingulate cortex (PCC; 1, −61, 38).

### Statistical Analyses

Two sample *t*-test was conducted in order to seek potential differences in FC measures between BD and HC groups (“ttest2” function). The results were corrected with the Benjamini and Hochberg (1995) False Discovery Rate correction (“fdr_bh” function) at *p* < 0.05 for all 55 connections, according to *N* × (*N*-1)/2 formula. Aforementioned connections were created from seven ROIs in SN and four ROIs in DMN. Only significant connections were then correlated with the SBQ-R score due to exploratory nature of this study.

## Results

**FIGURE 1:**
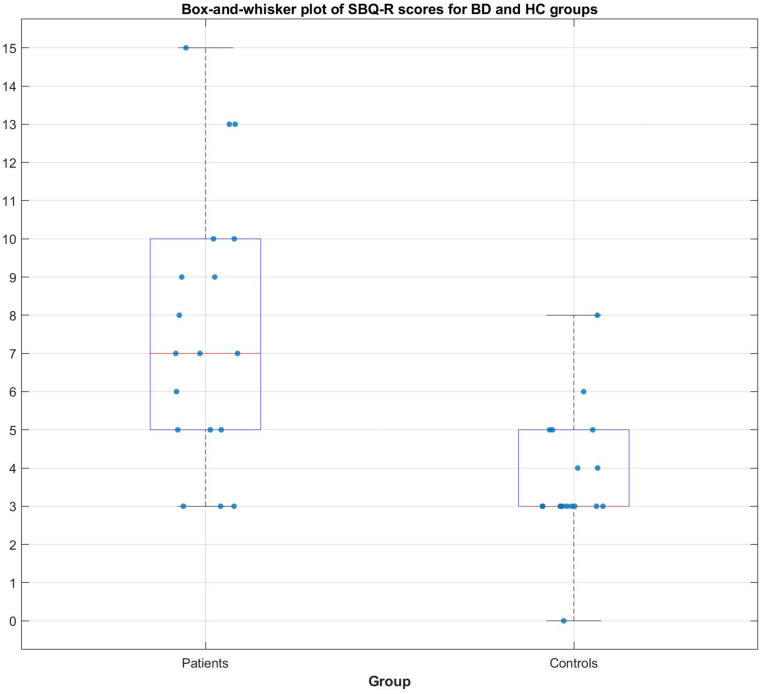
Distribution of SBQ-R score for BD and HC group.

**FIGURE 2:**
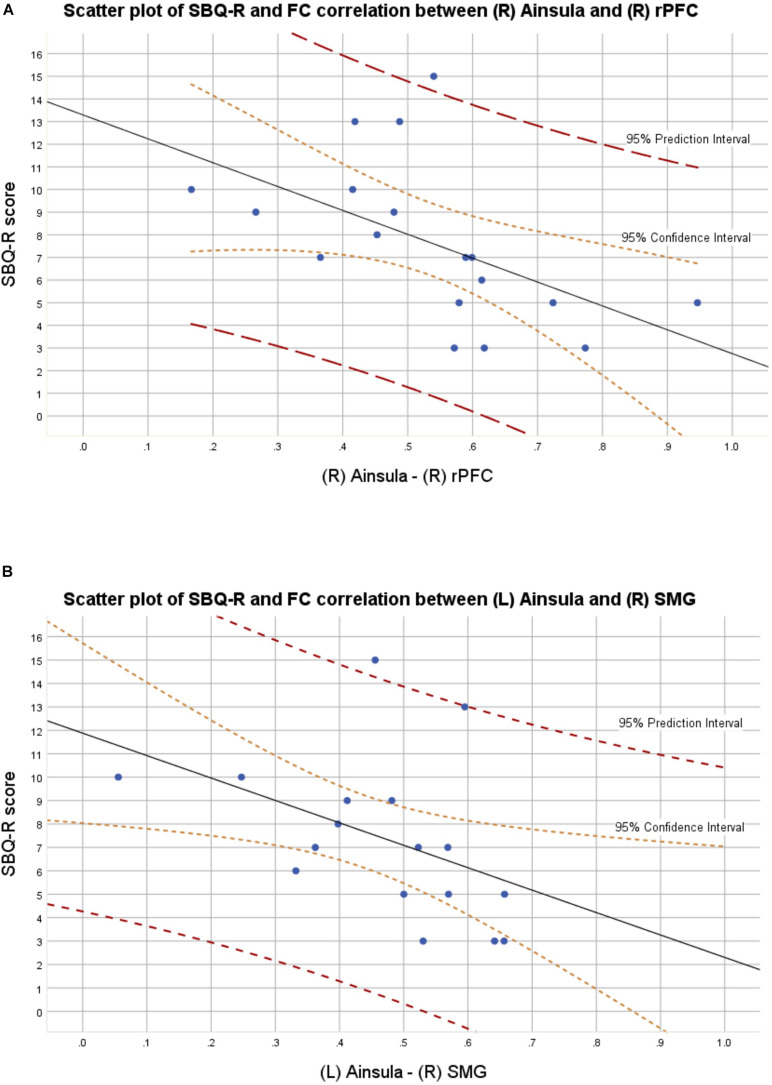
**(A)** Scatter plot with confidence and prediction intervals showing association between SBQ-R scores and raw functional connectivity values between right insula and right rostral prefrontal cortex in euthymic BD patients. **(B)** Scatter plot with confidence and prediction intervals showing association between SBQ-R scores and raw functional connectivity values between left insula and right supramarginal gyrus in euthymic BD patients.

**FIGURE 3:**
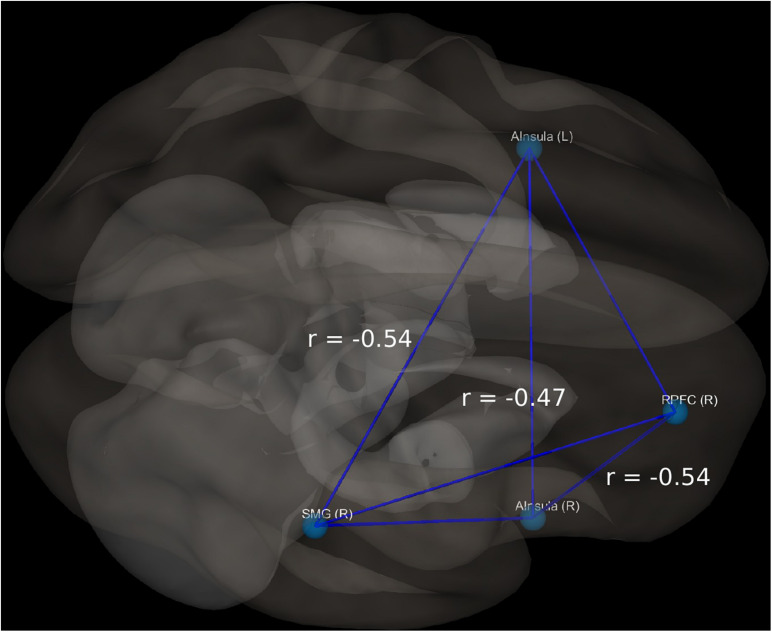
Visualization of significant differences in FC between patients and controls; r, correlation coefficient between selected structures and SBQ-R scale.

In the BD group, 9 out of 18 patients manifested significant current suicidal risk evaluated by the Suicide Behavior Questionnaire Revised (SBQ-R). There were no participants classified as having significant suicidal risk in HC group. The distribution of SBQ-R score in both groups is shown in Figure 1. Results of a two sample *t*-test showed significantly decreased functional connectivity (FDR corrected at *p* < 0.05) in BD patients in comparison to HC between the following regions of the SN: right anterior insular cortex and right rPFC [*t*_(__34__)_ = −3.2; *p* = 0.0045; Cohen’s *d* = 0.639]; left insula and right supramarginal gyrus [*t*_(__34__)_ = −3.9; *p* = 0.0007; Cohen’s *d* = 0.68] and right rPFC and right supramarginal gyrus [*t*_(__34__)_ = −2.2; *p* = 0.0344; Cohen’s *d* = 0.026]. Functional connectivity differences characterized with the largest effect sizes were visualized in Figure 2. All mentioned connections were negatively correlated with a high risk of suicide, evaluated using the SBQ-R (Figure 3): right anterior insular cortex and right rPFC (*p* = 0.0214, *r* = −0.54), left anterior insular cortex and right supramarginal gyrus (*p* = 0.0218, *r* = −0.54) and right rPFC and right supramarginal gyrus (*p* = 0.0469, *r* = −0.47). No significant differences were found between euthymic BD patients and HCs among regions involved in DMN.

## Discussion

The aim of our study was to investigate the baseline brain activity in euthymic BD patients in regard to suicide risk. In order to do that, a ROI-ROI functional connectivity approach based on rsfMRI data was used. Numerous previous studies indicate abnormal intrinsic functional connectivity among patients with euthymic BD patients. For instance, Korgaonkar et al. (2019) revealed significant hyper-connectivity in the DMN among euthymic BD subjects in comparison with HCs. Furthermore, according to Rey et al. (2016), euthymic participants exhibit decreased functional connectivity between the ACC and the PCC when compared to non-euthymic patients. Aforementioned results demonstrate an existing relationship between euthymic state and activity of both DMN and SN. Other studies associate activity of regions involved in these networks with suicide-related behaviors.

For instance, Reisch et al. (2010) found that the ACC and mPFC are active during recall of the suicidal episodes among participants who attempted suicide. Moreover possible association of disturbances within DMN and SN and vulnerability to suicidal behavior was also mentioned *inter alia* by Van Heeringen et al. (2014). A noteworthy fact is that negative self-concept is associated with suicidal behaviors (Bhar et al., 2008; Santos et al., 2009; Thompson, 2010), whereas regions which are considered to be a part of the DMN (Gusnard and Raichle, 2001; Raichle et al., 2001) are known to be involved in self-referential processing (Northoff and Bermpohl, 2004; Northoff et al., 2006).

Results of our study demonstrate decreased functional connectivity between (a) the right rPFC and right anterior insular cortex, (b) the right rPFC and the right supramarginal gyrus, as well as (c) the right supramarginal gyrus and left anterior insular cortex in euthymic BD patients in comparison with HCs. All aforementioned functional connections appeared to be negatively correlated with the risk of suicide. The above results support our second hypothesis assuming altered functional connectivity in SN and its relationship with suicide risk among euthymic BD patients in comparison to HC.

Anterior insular cortex (AI) holds responsibility for the overall integration of information relating to bodily states into higher-order cognitive and emotional processes (Gu et al., 2012). Furthermore, Thielscher and Pessoa (2007) revealed that the AI may be also important in the generation of perceptual choice, which is interesting in the light of strong evidence for working memory and decision making problems in BD patients during euthymia (Bhatia et al., 2018). Brass and Haggard (2007) demonstrate insula association with frustration, described as the affective consequence of canceling a motor intention. Their conclusions come in line with Craig (2009) results, indicating that insula is responsible for cognitive control and performance monitoring. Additionally, insula is crucial for self-recognition, risk evaluation as well as anticipation, which seem to be crucial processes for suicide-related behaviors (Northoff et al., 2006). Other significant region from our study – supramarginal gyrus – is thought to be essential in self-reference processes as well as overcoming emotional egocentricity bias in social judgments (Silani et al., 2013). In addition, it controls task switching, especially for response modality and stimulus-categorization switching (Philipp et al., 2013). The process of task switching is crucial in terms of general interpretation of a situation, therefore plays key role in suicide-related behaviors. Another important brain structure, which is rPFC, is supposed to play an enormous role in higher cognitive functions, thus its responsibility for episodic (Gilbert et al., 2006), prospective (Burgess et al., 2008), and retrospective memory (Burgess et al., 2003). What is more, it is involved in processes of learning from previous experiences. All of these functions are thought to be linked with suicide-related behaviors (Moore, 1997; Lemogne et al., 2006).

The strongest effect size of FC (functional connectivity) in our study was observed between right supramarginal gyrus and left anterior insular cortex (Cohen’s *d* = 0.68) as well as the right rPFC and right anterior insular cortex (Cohen’s *d* = 0.639) which may indicate that individuals with high suicide risk potentially demonstrate difficulties in learning from previous mistakes as well as interpretation of various situations, what can lead to risky, as well as adverse behaviors. On top of that, changed activity of brain structures which are considered to be responsible for prospective memory, suggest that euthymic BD patients may manifest problems with planning and meaning of sense. This conclusion is corresponding with Moore (1997) describing difficulties experienced by patients characterized with a high suicide risk. Moreover, our results confirm that euthymic BD patients demonstrate abnormal changes in intrinsic brain networks, especially related with self-referential mental activity (Marchand et al., 2013), whereas negative self-concept may be associated with suicidal behaviors. These results, combined with cognitive inflexibility in terms of ambiguous live circumstances, may be valuable predictors for suicidal ideation and suicide-related behaviors (Miranda et al., 2012).

Conducted study had some limitations. First, the robustness of our results could be improved with an increased sample size. What is more, the euthymic BD group was heterogeneous in regard to the type of the disorder. Regardless, there is a strong need to improve our knowledge about the risk of suicide among BD patients, especially during euthymic phase and to our best knowledge this is the first rsfMRI study covering that topic. Results presented in this study appeared to be significant and provide ground for future research with a larger sample size. However results considering FC between right rPFC and right supramarginal gyrus with considerably small effect size of *d* = 0.026 should be interpreted with caution. Further examination of the relationship between resting state activity and suicidal behavior is promising way, which may enable to develop novel diagnostic methods.

## Data Availability Statement

The raw data supporting the conclusions of this article will be made available by the authors, without undue reservation.

## Ethics Statement

The studies involving human participants were reviewed and approved by the Jagiellonian University Bioethics Committee. The patients/participants provided their written informed consent to participate in this study.

## Author Contributions

AMS: research design, conceptualization, methodology, data collection, fMRI data analysis, writing – original draft, and writing – review and editing. BB: conceptualization, methodology, data collection, fMRI data analysis, and writing – review and editing. TM: conceptualization, supervision, and writing – review and editing. MF: writing – review and editing, and supervision. DD and MS: patient recruitment and supervision. AT and AK: patient recruitment and writing – review and editing. AB: clinical evaluation of MRI data. AC: patient recruitment, data collection, project administrator, writing – review and editing, and supervision. All authors contributed to the article and approved the submitted version.

## Conflict of Interest

The authors declare that the research was conducted in the absence of any commercial or financial relationships that could be construed as a potential conflict of interest.
